# Acute myocardial infarction induced by avatrombopag: a case report

**DOI:** 10.3389/fphar.2025.1618693

**Published:** 2025-08-22

**Authors:** Gou Junqi, Liu Chaohui, Lang Mingjian, Yao Fengyou

**Affiliations:** Department of Cardiology, Geriatric Diseases Institute of Chengdu/Cancer Prevention and Treatment Institute of Chengdu, Chengdu Fifth People’s Hospital (The Second Clinical Medical College, Affiliated Fifth People’s Hospital of Chengdu University of Traditional Chinese Medicine), Chengdu, China

**Keywords:** avatrombopag, acute myocardial infarction, coronary thrombosis, immune thrombocytopenia, thrombopoietin receptor agonists

## Abstract

**Background:**

Avatrombopag, a thrombopoietin receptor agonist (TPO-RA), is used for immune thrombocytopenia (ITP) but confers thrombotic risks. Acute myocardial infarction (AMI) as an adverse event is underreported.

**Summary:**

A 58-year-old female with steroid-refractory ITP developed ST-elevation myocardial infarction (STEMI) 5 days after initiating avatrombopag monotherapy (20 mg/day). She had no history of traditional cardiovascular risk factors. Her platelet count increased from two to 122 × 10^9^/L before AMI. Coronary angiography revealed thrombotic occlusion of the left ventricular posterior branch, treated with thrombus aspiration. Dual antiplatelet therapy was initiated, and avatrombopag was discontinued. The patient was discharged on day 10 post-AMI. At the 14-day follow-up, thrombocytopenia recurred (platelets 18 × 10^9^/L), requiring avatrombopag re-initiation alongside aspirin. No further thrombosis occurred.

**Conclusion:**

Avatrombopag monotherapy may induce rapid coronary thrombosis. Prophylactic antiplatelet therapy and maintaining platelets at 50–150 × 10^9^/L are critical during TPO-RA treatment. This case highlights the need for thrombotic risk assessment before TPO-RA initiation.

## 1 Introduction

Immune thrombocytopenia (ITP) is a common bleeding disorder clinically characterized by isolated thrombocytopenia in the peripheral blood without identifiable causes. The pathogenesis of ITP remains incompletely understood, but it is widely attributed to immune-mediated excessive platelet destruction or insufficient platelet production. Certain viral infections may trigger or exacerbate ITP, and a subset of patients may exhibit genetic susceptibility (Trisolini et al., 2024). Pharmacological treatment remains the mainstay of therapy to effectively relieve symptoms. Commonly used agents include glucocorticoids, intravenous immunoglobulin, anti-D immunoglobulin, rituximab, splenectomy, and thrombopoietin receptor agonist (TPO-RA) (Bolton-Maggs and George, 2021). Avatrombopag is a second-generation, oral, non-peptide TPO-RA that primarily targets the thrombopoiesis pathway. It selectively activates thrombopoietin receptors in bone marrow, promoting the proliferation and differentiation of megakaryocytes via the Janus Kinase 2 (JAK2)/Signal Transducer And Activator Of Transcription 5 (STAT5) and Mitogen-Activated Protein Kinase (MAPK)/Extracellular (ERK) signaling pathways, ultimately increasing platelet counts (Markham, 2021). In May 2018, avatrombopag was approved by the FDA for ITP treatment. Clinical trials have demonstrated that avatrombopag significantly increases platelet counts and maintains them at effective levels. Despite its efficacy in treating thrombocytopenic conditions, avatrombopag is associated with adverse effects, including thrombotic or thromboembolic events, headache, fatigue, and gastrointestinal toxicity. While thrombotic risks of TPO-RA are documented, AMI cases remain underreported—only 13 FDA-reported avatrombopag-linked AMI cases exist (Table 1) (Xue et al., 2024). This gap underscores the need to characterize TPO-RA-induced coronary thrombosis. We present a case of rapid-onset STEMI following avatrombopag monotherapy, aiming to: (1) establish causality via temporal association and exclusion of confounders; (2) propose thromboprophylaxis protocols for high-risk patients.

**TABLE 1:** FDA faers reports of avatrombopag-associated AMI (2008–2023).

Characteristic	Present case	FDA reports (n = 13)	Comment
Reported sex	Female	8 Male, 5 Female	Male predominance
Age (years)	58	Median: 68 (Range: 44–83)	Older population
Indication	ITP	11 Chronic Liver Disease (CLD), 2 ITP	CLD most common
Daily dose	20 mg	20–40 mg	Similar dosing
Time to onset (days)	5	Median: 7 Range: 1–42	Rapid onset (≤7) common
Platelet count at Event (×10^9^/L)	122	Not consistently reported	Not reliable predictor of thrombosis
Outcome	Recovered	9 Recovered, 3 Fatal, 1 Unknown	Highlights severity

## 2 Case presentations

A 58-year-old female presented to the hematology department on 8 December 2024, with a 4-month history of thrombocytopenia. She was initially diagnosed with ITP in May 2024 at our institution, she showed poor response to oral methylprednisolone. Due to steroid-refractoriness and the patient’s preference against splenectomy. In July 2024, treatment was switched to avatrombopag 20 mg once daily (discontinued after 14 days when platelets reached 300 × 10^9^/L) (Chinese Society of Hematology and Thrombosis and Hemostasis Group, 2023a) plus aspirin for thrombosis prophylaxis (initiated when platelets reached 30–70 × 10^9^/L) (Table 2) (Chinese Society of Hematology and Thrombosis and Hemostasis Group, 2023b). In September 2024, she was rehospitalized for severe thrombocytopenia (3 × 10^9^/L) and received avatrombopag 20 mg once daily (discontinued after 14 days when platelets rose to 254 × 10^9^/L) with continued aspirin prophylaxis. On 8 December 2024, the patient was readmitted for severe thrombocytopenia with a platelet count of 2 × 10^9^/L. Her medical history was unremarkable except for chronic hepatitis B managed with long-term oral entecavir. On admission, her vital signs were as follows: temperature: 36.9 °C; pulse: 89 beats/min; respiratory rate: 16 breaths/min; blood pressure:125/93 mmHg. She was 170 cm tall, weighed 60 kg, and had a body mass index (BMI) of 20.7 kg/m^2^. Physical examination was unremarkable. Laboratory tests revealed severe thrombocytopenia with a platelet count of 2 × 10^9^/L (reference range: 100–300 × 10^9^/L). Fecal occult blood was weakly positive (±), and urinary occult blood was 1+. N-terminal Pro-brain Natriuretic Peptide (NT-proBNP), D-dimer, cardiac enzymes, liver and renal function, lipid profile, thyroid function, electrolytes, and glycated hemoglobin were within reference ranges. Electrocardiography indicated sinus bradycardia with left anterior fascicular block. Transthoracic echocardiography, abdominal ultrasonography, and chest computed tomography showed no significant abnormalities.

**TABLE 2:** Chronological summary of Patient’s clinical course and key parameters.

Parameter	Avatrombopag First use	Admission	AMI onset	Discharge	Follow up
Platelet Count (×10^9^/L)	12	2	122	310	18
cTnI (ng/mL)	—	<0.1	4.47	6.53	0.43
MYO (ng/mL)	—	3.47	295	43.6	7.92
CK-MB (ng/mL)	—	2.75	4.28	4.72	2.64
NT-proBNP (pg/mL)	202.05	130.57	627	1708	942.4
Occult Blood Tests	—	Urine:1+ Stool:±	—	—	—
ECG	Left anterior fascicular block	Unchanged	ST-segment elevation	ST-segment depression	Left anterior fascicular block
Coronary angiographic	—	—	Thrombotic occlusion	—	—
Medications	Avatrombopag Aspirin	Avatrombopag Dexamethasone	Emergency PCI	Aspirin Ticagrelor	Avatrombopag Aspirin
Antithrombotic Strategy	Antiplatelet combo restarted	No antiplatelet agent	Avatrombopag D/C	Dual antiplatelet	Antiplatelet combo restarted

## 3 Treatment course

**FIGURE 1:**
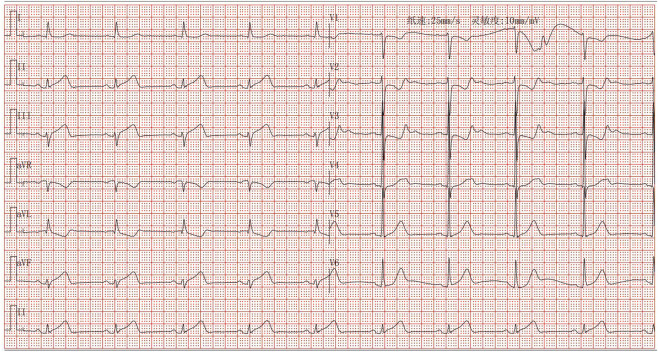
Electrocardiogram of acute inferior ST-segment elevation myocardial infarction.

**FIGURE 2:**
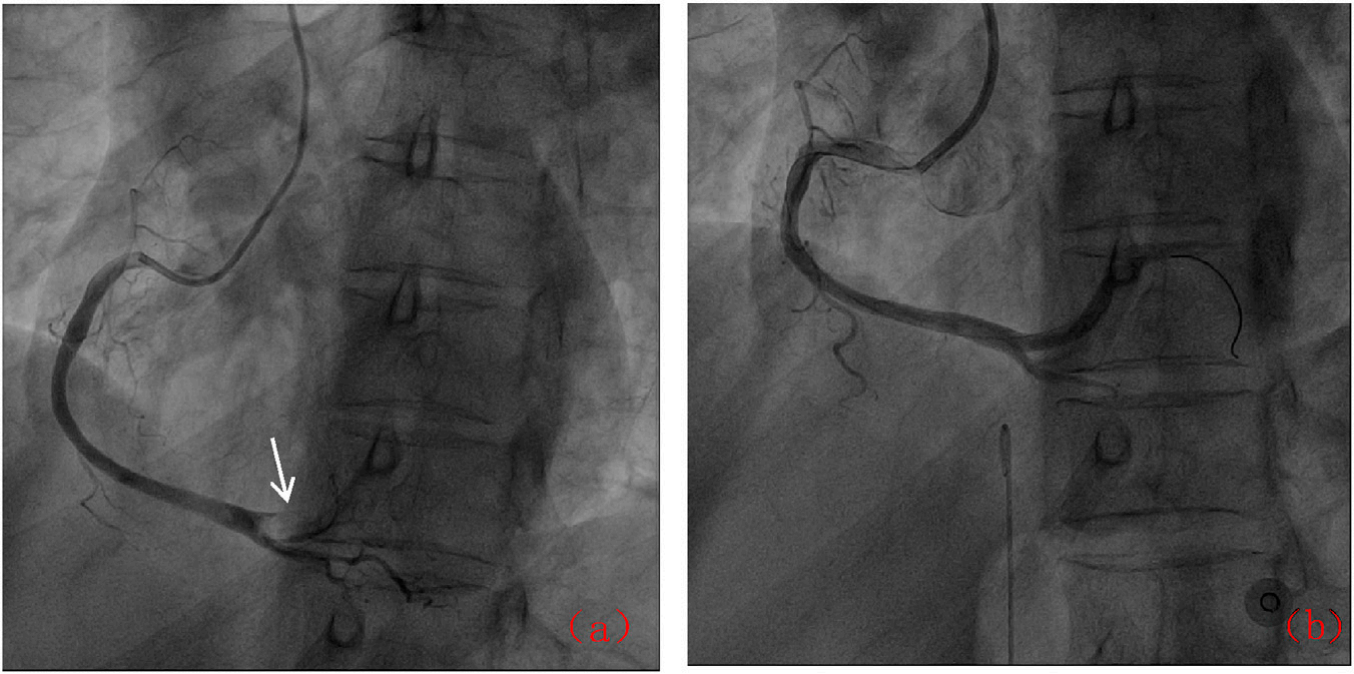
Coronary angiographic results: **(a)** Acute occlusion of the posterior left ventricular branch (arrow); **(b)** Post-thrombus aspiration showing restored flow.

Following admission, the patient was administered avatrombopag 20 mg once daily and intravenous dexamethasone sodium phosphate 20 mg once daily to elevate platelet levels (aspirin was not co-administered due to positive urinary occult blood). A 40 mg daily dose of omeprazole injection was administered to prevent acute gastric mucosal lesions. A single therapeutic unit of apheresis platelets was transfused. On 12 December 2024, the platelet count was reassessed and found to be 122 × 10^9^/L. Later that day, the patient developed chest pain. Electrocardiography revealed sinus bradycardia and ST-T changes, with ST-segment elevation of 0.05–0.10 mV in leads II, III, aVF, and V7-9, and biphasic T waves in V2-3 (Figure 1). Laboratory tests showed elevated myocardial markers: Myoglobin (MYO): 295 ng/mL (reference range: <50 ng/mL), Cardiac Troponin I (cTnI): 4.47 ng/mL (reference range: <0.1 ng/mL), NT-proBNP: 627 pg/mL (reference range: <900 pg/mL), and Creatine Kinase-Myocardial Band (CK-MB): 4.28 ng/mL (reference range: <5 ng/mL). Emergency coronary angiography revealed acute occlusion at the origin of the left ventricular posterior branch (Figure 2). A large thrombus was aspirated from the coronary artery, and subsequent angiography showed no fixed stenosis in the left ventricular posterior branch, indicating coronary artery thrombosis. Postoperatively, the patient was treated with dual antiplatelet therapy consisting of aspirin enteric-coated tablets (100 mg nightly) and ticagrelor (90 mg twice daily). Additional therapies included atorvastatin calcium tablets (20 mg nightly) for lipid lowering, pantoprazole sodium tablets (40 mg once daily) for gastric mucosal protection, low-molecular-weight heparin (6000 IU subcutaneously every 12 h) for anticoagulation. Her chest pain resolved completely by the second postoperative day. Her history included the use of avatrombopag in combination with aspirin without thrombotic events. In this hospitalization, aspirin was withheld due to occult bleeding. Coronary thrombosis occurred after 4 days of avatrombopag administration. The strikingly rapid onset of AMI (5 days post-avatrombopag initiation) coincided precisely with the peak platelet rise, reinforcing a direct link. Following consultation with hematology specialists, coronary artery thrombosis was considered potentially related to avatrombopag use. Discontinuation of avatrombopag was recommended, and dual antiplatelet therapy with aspirin enteric-coated tablets and ticagrelor was continued, with close monitoring of platelet count. On 17 December 2024, platelet count was 310 × 10^9^/L, cTnI was 6.53 ng/mL, and NT-proBNP was 1,708 pg/mL. Fourteen days after discharge, the outpatient platelet count was 18 × 10^9^/L. Aspirin and ticagrelor were discontinued, and the oral administration of 20 mg once daily of avatrombopag and 100 mg nightly of aspirin was resumed. As of the latest follow-up, no further thrombosis events have occurred.

## 4 Discussion

### 4.1 Analysis of AMI etiology in this patient

This case involved a 58-year-old female patient with no history of cardiovascular disease risk factors. The 10-year risk of developing atherosclerotic cardiovascular disease in this patient was classified as low. After being diagnosed with ITP and failing to respond to corticosteroid therapy, the patient received avatrombopag for platelet elevation. No thrombotic events occurred during the first or second course of combined avatrombopag and aspirin therapy. However, during this hospitalization, avatrombopag was administered as monotherapy due to suspected urinary bleeding, and acute inferior STEMI occurred after 5 days of avatrombopag monotherapy. Subsequently, avatrombopag was discontinued, and standard secondary prevention therapy for coronary artery disease was initiated. Fourteen days after discharge, her platelet count was 18 × 10^9^/L, and avatrombopag was reintroduced alongside aspirin. During follow-up, the avatrombopag dosage was dynamically adjusted (initiated at 20 mg daily, then tapered when platelets exceeded 50 × 10^9^/L to maintain levels between 50–150 × 10^9^/L), and no further thrombotic events occurred. Naranjo algorithm assessment for coronary thrombosis associated with avatrombopag in this case yielded a score of 6, indicating a probable causal relationship. Differential diagnoses including spontaneous coronary artery dissection (ruled out by IVUS), coronary spasm (unresponsive to nitroglycerin), and paradoxical embolism (no DVT/shunt) were rigorously excluded. The aspirated thrombus and close temporal association (5 days post-avatrombopag initiation) strongly support drug-induced coronary thrombosis. Notably, adverse reactions listed in the drug’s prescribing information include arterial and venous thrombosis or thromboembolism, with a reported incidence of 7%. While this case highlights the potential thrombotic risks of avatrombopag, the broader implications of TPO-RAs in inducing thrombosis require further exploration. The following section examines the mechanisms and clinical challenges of TPO-RAs-associated thrombotic events.

### 4.2 Thrombosis/embolism induced by TPO-RAs

ITP is the most common bleeding disorder in hematology (Trisolini et al., 2024), is primarily caused by immune dysregulation that leads to both increased platelet destruction and reduced platelet production. Pharmacological treatment remains the mainstay of therapy to effectively relieve the symptoms. Commonly used agents include glucocorticoids, intravenous immunoglobulin, and TPO-RAs. TPO-RAs act by activating thrombopoietin receptors, thereby promoting the division of megakaryocytes in the bone marrow and the release of platelets into circulation. These agents are indicated for thrombocytopenia associated with chemotherapy, ITP, aplastic anemia, and chronic liver disease (Guillet et al., 2023). Clinically available TPO-RAs include non-peptide agents (such as eltrombopag, hetrombopag, avatrombopag, and lusutrombopag) and peptide mimetics (such as romiplostim). Avatrombopag binds to the TPO receptor*’*s transmembrane domain, activating a signaling cascade. This promotes megakaryocyte proliferation and differentiation, ultimately increasing platelet counts. Notably, avatrombopag neither stimulates the production of anti-TPO antibodies nor competes with endogenous TPO for binding sites. Instead, it exerts an additive effect with endogenous TPO in promoting platelet production. Romiplostim, on the other hand, is a recombinant Fc-peptide fusion protein produced using DNA technology. It consists of two identical subunits, each comprising an IgG1 Fc domain fused to a 14-amino-acid peptide fragment. Romiplostim exhibits a high affinity for the TPO receptor and directly competes with TPO for receptor binding. Upon binding, it activates JAK2, STAT5, and MAPK signaling pathways, thereby stimulating megakaryocyte proliferation and platelet production (Soff et al., 2024).

With the widespread clinical application of TPO-RAs, reports of drug-associated adverse events have gradually increased, with thrombosis being one of the serious adverse reactions (Abdelsamia et al., 2023). The risk of thrombosis in patients with ITP is multifactorial. ITP-related factors include the presence of immature, hyperactive platelets, circulating megakaryocytes, elevated levels of prothrombin microparticles, altered von Willebrand factor activity, and the effects of autoantibodies on endothelial surfaces. Additionally, there are complex interactions between ITP-specific factors and individual risk factors such as obesity, diabetes, hypercholesterolemia, hypertension, smoking, atrial fibrillation, valvular disease, and coronary artery disease. TPO-RA therapy modulates these interactions promotes platelet activation,and sustains a procoagulant state by increasing platelet microparticle formation and enhancing the expression of glycoprotein VI and P-selectin on the platelet surface (Napolitano et al., 2023).

Although TPO-RAs may improve bleeding risk by increasing platelet count, excessive platelet count or hyperfunction can disrupt the coagulation-anticoagulation balance (van Dijk et al., 2021). The research results indicate that the incidence of arteriovenous thrombotic events in ITP patients receiving TPO-RAs treatment is 2–3 times higher than that in patients not receiving TPO-RAs treatment (Tjepkema et al., 2022), and platelet count is not linearly correlated with thrombotic events (van Dijk et al., 2021). Thrombotic events are also observed in both low and reference range platelet counts (van Dijk et al., 2021). These mechanistic insights underscore the clinical dilemma: while TPO-RAs effectively elevate platelet counts, their prothrombotic effects may offset the benefits in certain patients. Notably, this patient lacked traditional cardiovascular risk factors (such as hypertension and diabetes), yet developed AMI shortly after initiating avatrombopag monotherapy, which strongly suggests that the mechanism of drug-induced thrombosis is dominant. This phenomenon is consistent with the temporal characteristics of myocardial infarction cases related to avatrombopag in FDA adverse event reports (Xue et al., 2024).

The necessity of combining anticoagulant or antiplatelet therapy with TPO-RAs remains controversial. China’s guidelines suggest (Chinese Society of Hematology and Thrombosis and Hemostasis Group, 2023b) a platelet count of 30–50 × 10^9^/L, which can be combined with antiplatelet or anticoagulant drugs; Platelet count>50–70 × 10^9^/L, combined antiplatelet and anticoagulant drugs are required. The temporal pattern of thrombotic events following TPO-RAs therapy remains poorly characterized. Current guidelines recommend (Chinese Society of Hematology and Thrombosis and Hemostasis Group, 2023a): Close monitoring of platelet counts and thrombotic markers during the initial treatment phase, with the lowest effective dose to maintain platelets within 50–150 × 10^9^/L; Dose reduction strategies prioritizing decreased administration frequency (e.g., extending intervals between doses) over abrupt discontinuation; Post-adjustment evaluation after a 2-week observation period to assess hematologic response before further dose modifications. If platelet counts exceed 250 × 10^9^/L, discontinuation of TPO-RAs therapy is advised to mitigate the risk of bleeding or thromboembolic events. After discontinuation, platelet levels and thrombotic events should be monitored closely (at least twice weekly) and therapy may be resumed if a downward trend in platelet counts is observed. The optimal timing for discontinuing TPO-RAs in ITP patients remains under investigation. United Kingdom consensus guidelines suggest tapering may be attempted in patients who have received TPO-RAs therapy for >6 months with sustained platelet counts >50 × 10^9^/L, no bleeding events, and low bleeding risk for 6 months (Cuker et al., 2022). The U.S. expert consensus recommends attempting a tapering strategy for patients with platelet counts >150 × 10^9^/L, no major hemorrhage, low trauma risk, and no requirement for aggressive therapy (Cuker et al., 2020).

## 5 Conclusion

This case illustrates avatrombopag monotherapy*’*s potential to induce rapid coronary thrombosis, even without traditional cardiovascular risks. Prophylactic antiplatelet therapy is imperative during TPO-RA treatment, with platelet targets of 50–150 × 10^9^/L. Multidisciplinary risk assessment (incorporating platelet function tests, cardiovascular history, and pharmacovigilance timelines) should guide therapeutic decisions. Future studies should validate risk-stratification tools (e.g., platelet microparticle assays) and explore optimal antithrombotic regimens.

## Data Availability

The original contributions presented in the study are included in the article/Supplementary Material, further inquiries can be directed to the corresponding author.

## Abbreviations

TPO-RA, Thrombopoietin Receptor Agonist; ITP, Immune Thrombocytopenia; AMI, Acute Myocardial Infarction; STEMI, ST-elevation Myocardial Infarction; MYO, Myoglobin; NT-proBNP, N-terminal Pro-brain Natriuretic Peptide; cTnI, Cardiac Troponin I; CK-MB, Creatine Kinase-Myocardial Band; JAK2, Janus Kinase 2; STAT5, Signal Transducer And Activator Of Transcription 5; MAPK, Mitogen-Activated Protein Kinase; ERK, Extracellular; CLD, Chronic liver disease.
