# Supplementary material for: Acute myocardial infarction induced by avatrombopag: a case report
Source: Front Pharmacol. 2025 Aug 22;16:1618693. doi: 10.3389/fphar.2025.1618693 (PMC12421002; doi:10.3389/fphar.2025.1618693)
Supplement: Supplementary file 1 [file Supplementaryfile1.docx]

# ****2013 CARE Checklist****

**1、Title** – **Compliance:** "Acute Myocardial Infarction Induced by Avatrombopag: A Case Report" clearly states the intervention (avatrombopag) and study design (case report), adhering to CARE guidelines.

**2、Key Words** – Avatrombopag, acute myocardial infarction, coronary thrombosis, immune thrombocytopenia, case report.

**3、Abstract** – (structured or unstructured)

**3a Introduction** – Avatrombopag, a second-generation thrombopoietin receptor agonist (TPO-RA), is effective for immune thrombocytopenia (ITP) but carries a risk of thrombotic events. This case is the first to report acute coronary thrombosis during avatrombopag monotherapy, highlighting the need for vigilance regarding TPO-RA-related thrombosis.

3b **Clinical Findings:**A 58-year-old female with ITP and no cardiovascular risk factors developed acute inferior ST-segment elevation myocardial infarction (STEMI) five days after initiating avatrombopag monotherapy (previously combined with aspirin without complications).

**3c. Interventions/Outcomes:**Emergency coronary angiography revealed acute occlusion of the left ventricular posterior branch. Thrombus aspiration and dual antiplatelet therapy (aspirin + ticagrelor) resolved symptoms. Platelet counts fluctuated post-discharge, but no recurrent thrombosis occurred.

**3d. Conclusion:**This case underscores the thrombotic risk of TPO-RAs, emphasizing the need for risk stratification and close monitoring during therapy.

**4、Introduction** – **Compliance:** Explains ITP pathophysiology, limitations of conventional therapies, and rationale for TPO-RAs. Cites FDA approval data and clinical trials supporting avatrombopag use, while emphasizing its thrombotic risks. References align with current literature.

**5、Patient Information**

**5a.Demographics:** 58-year-old female, de-identified (no name/location).

**5b. Symptoms:** Severe thrombocytopenia (platelet count 2×10⁹/L), chest pain, and STEMI signs (ST-segment elevation).

**5c. History:**Chronic hepatitis B (managed with entecavir), ITP diagnosed in May 2024, no cardiovascular comorbidities.

**5d. Past Interventions:**Initial treatment with methylprednisolone (poor response), followed by avatrombopag + aspirin (platelet stabilization).

**6、 Clinical Findings** –

**Physical Exam:** Normal cardiac auscultation, no murmurs, clear lungs.

**Diagnostics:**ECG: ST-segment elevation in inferior leads (Fig. 1).Coronary angiography: Acute occlusion of the left ventricular posterior branch (Fig. 2).Lab: Platelet count 2×10⁹/L, elevated cardiac biomarkers (TnI: 4.47 ng/mL).

1. **Timeline** – **May 2024:** ITP diagnosis; initiated methylprednisolone (ineffective).

**July 2024:** Switched to avatrombopag + aspirin (platelet count stabilized).

**December 8, 2024:** Hospitalized for severe thrombocytopenia; avatrombopag monotherapy started.

**December 12, 2024:** Developed STEMI; thrombus aspiration performed.

**December 18, 2024:** Discharged on dual antiplatelet therapy.

**Follow-up (14 days):** Platelet count dropped to 18×10⁹/L; avatrombopag + aspirin resumed.

**Follow-up (After 6 months):** Sustained hemodynamic/functional improvements.

**8、Diagnostic Assessment**

**8a. Testing:**ECG, coronary angiography, platelet count, cardiac biomarkers (TnI, NT-proBNP).

**8b. Challenges:**Aspirin contraindication due to occult bleeding risk necessitated avatrombopag monotherapy.

**8c. Diagnosis:**Avatrombopag-induced acute coronary thrombosis (no fixed stenosis on angiography).

**8d. Prognosis:**Favorable short-term outcome post-intervention; long-term monitoring required for platelet and thrombosis risks.

**9、Therapeutic Intervention**

**9a. Type:**Acute phase: Thrombus aspiration, dual antiplatelet therapy (aspirin + ticagrelor).Maintenance: Avatrombopag + aspirin (adjusted based on platelet counts).

**9b. Administration:**Avatrombopag 20 mg daily; ticagrelor 90 mg bid; aspirin 100 mg qn.

**9c. Changes:**Avatrombopag temporarily discontinued post-STEMI; resumed with aspirin after platelet drop.

**10、Follow-up and Outcomes**

**10a.Outcomes:**Patient-reported: Resolution of chest pain.

Clinician-assessed: No recurrent thrombosis, platelet stabilization.

**10b. Tests:**Platelet count: 310×10⁹/L post-procedure, 18×10⁹/L at 14-day follow-up.

**10c. Adherence:**Regular follow-up; compliance with antiplatelet therapy confirmed.

**10d. Adverse Events:**Acute STEMI attributed to avatrombopag; no further complications reported.

**11、Discussion**

**11a.Strengths/Limitations:**Strengths:First report linking avatrombopag monotherapy to coronary thrombosis; comprehensive diagnostic workup.Limitations: Single-case design; lack of long-term data.

**11b. Literature Comparison:**Aligns with FDA reports (84 thrombotic events, including 13 myocardial infarctions) and meta-analyses on TPO-RA risks.

**11c. Rationale:**TPO-RAs enhance platelet activation and microparticle formation, promoting a prothrombotic state.

**11d. Take-away:**Clinicians must weigh thrombotic risks against benefits when prescribing TPO-RAs, especially in monotherapy settings.

1. **Patient Perspective** –

**Missing:** Clinicians must weigh thrombotic risks against benefits when prescribing TPO-RAs, especially in monotherapy settings.

**Implied:** Symptom improvement and adherence to therapy suggest a positive perspective.

**Informed Consent** – **Compliance:** Explicitly stated: "Written informed consent was obtained from the patient to publish this case report."
